# Localized Cutaneous Versus Deep Sporotrichosis at Hospital das Clínicas, UFMG, Belo Horizonte, Brazil: A Cohort of 213 Culture-Confirmed Cases, 2011 to 2026

**DOI:** 10.3390/jof12090685

**Published:** 2026-09-11

**Authors:** Helena Duani, Frederico Figueiredo Amâncio, Alexandre Soares Bifano, Vitória Baltazar Mattos, Kaique Veloso Silva, Beatriz Vieira Melo, Heloisa Filipe Pascoal, Rafael Lima Sendin, Marcus Vinicius Carvalho Akerman, Felipe Torreão Corrêa Thiemann, Giulia Bravim Gonçalves, Adriana Cristina Camargos de Rezende, Leandro Cesar da Silva, Shinfay Maximilian Liu, Marise Oliveira Fonseca

**Affiliations:** 1 Infectious Diseases Service, Department of Internal Medicine, Faculty of Medicine, Universidade Federal de Minas Gerais, UFMG, Belo Horizonte 30130-100, Minas Gerais, Brazil; 2 Postgraduate Program in Infectious Diseases and Tropical Medicine, Faculty of Medicine, Universidade Federal de Minas Gerais, UFMG, Belo Horizonte 30130-100, Minas Gerais, Brazil; 3 Santa Casa de Patrocínio, Patrocínio 38740-000, Minas Gerais, Brazil; 4 Microbiology Laboratory, Hospital das Clínicas, Universidade Federal de Minas Gerais, UFMG, Belo Horizonte 30130-100, Minas Gerais, Brazil

**Keywords:** sporotrichosis, *Sporothrix brasiliensis*, itraconazole, implantation mycosis, cohort, Brazil

## Abstract

Background: The zoonotic epidemic of sporotrichosis caused by *Sporothrix brasiliensis* has expanded throughout Brazil and other countries, with a rise in deep forms of disease. Detailed comparative data on the presentation, treatment and outcomes of cutaneous and deep disease are needed to follow how Brazilian cohorts are evolving. Methods: The study involves a retrospective observational cohort of patients with sporotrichosis treated at a quaternary university hospital in Belo Horizonte between 2011 and 2026, restricted to culture-positive cases. Localized cutaneous disease was compared with deep infection using logistic regression adjusted for age and sex. Results: Of 313 patients, 213 had a positive culture. The median age was 41 years, and 54.0% were women. Lymphocutaneous disease predominated (54.0%), and contact with cats was reported by 53.1%. The median treatment duration was 150 days. Among the 101 patients with a defined outcome, 97 were cured (96.0%), with no documented treatment failure. All four sporotrichosis-related deaths occurred in the deep infection group, which was independently associated with comorbidity (OR 9.69; 95% CI 2.52 to 37.23) and an immunosuppressive condition (OR 6.61; 95% CI 1.89 to 23.09). Conclusions: Localized cutaneous sporotrichosis was cured in nearly all the patients with documented follow-up, whereas mortality was confined to deep infection associated with comorbidities and immunosuppression.

## 1. Introduction

Sporotrichosis is one of the most frequent implantation mycoses worldwide, caused by thermodimorphic fungi of the genus *Sporothrix*, and is most prevalent in tropical and subtropical areas [1]. Infection results from traumatic inoculation of the fungus into the skin, classically from soil, plants or decaying organic matter, thereby making the disease an occupational hazard for almost a century [2]. Outside South America this remains the predominant presentation, caused mainly by *S. schenckii* and *S. globosa* [3].

This pattern changed at the end of the 1990s. Zoonotic transmission from cats infected with *Sporothrix brasiliensis*, an emerging species first described in Brazil and later in other South American countries, became the main mechanism in the country, moved the disease into the urban environment and began to affect mainly adult women who care for the animals at home, with disease also occurring in children [4,5,6]. Brazil is currently the epicenter of the ongoing epidemic [7]. Cat-transmitted disease has also been described in Asian countries, caused by *S. schenckii* [8,9].

The usual presentation is cutaneous, in the fixed cutaneous and lymphocutaneous forms, with good prognosis and response to itraconazole [10]. The expansion of the epidemic caused by *S. brasiliensis*, a more virulent species, has been accompanied by an increase in deep forms, with osteoarticular, pulmonary and central nervous system involvement and disseminated disease, which require prolonged treatment and are associated with functional sequelae and mortality [11,12,13,14,15,16,17]. These forms occur mainly in patients with comorbidities, alcohol use or immunosuppression and are often recognized late [18,19,20].

Sporotrichosis is described by the Brazilian Ministry of Health as the most prevalent implantation mycosis in the country, occurring in all regions. The true magnitude of the problem, however, remains unknown: the disease was only added to the National List of Notifiable Diseases in March 2025, and surveillance of endemic mycoses is expected to be implemented in all federal units by 2027 [21]. State and municipal series show consistent growth: in the state of São Paulo, 7834 cases were notified between 2011 and 2025, of which 6784 were in the last five years [22]; in Curitiba the incidence of *S. brasiliensis* infection increased steadily between 2011 and 2022 [23]; and, in Pernambuco, sporotrichosis became the second leading cause of hospital admission for fungal disease between 2016 and 2024 [24].

In Belo Horizonte, human sporotrichosis has been a notifiable disease at the municipal level since 2018. Between 2016 and 2026, 1,262 cases were confirmed among the residents of the municipality, with sustained growth over the period: 15 cases in 2016, 94 in 2020, 203 in 2023, 227 in 2024 and 380 in 2025. Women account for 64% of the cases and 67% occur between 20 and 69 years of age. Among the identified exposures, cats were involved in 74.8% and dogs in 15.4%, and the most frequent mechanism was scratching, followed by biting and contact with skin lesions of the animal [25]. The expansion of animal and human disease in the state since 2016 has been documented in molecular and epidemiological studies [26,27].

Treatment is prolonged, with a recommended duration of about four months for cutaneous forms and more than six months for deep forms [21], which raises issues of adherence, tolerability and laboratory monitoring. Clinical knowledge about the disease in Brazil derives largely from a single referral institution in Rio de Janeiro, the source of the largest series on severe forms, including disease in people living with HIV, bone sporotrichosis and pulmonary disease [12,13,18]. Descriptions of culture-confirmed cohorts from other regions, covering the full spectrum of disease and reporting actual treatment duration, tolerability of prolonged antifungal therapy and outcome by clinical form, are lacking. This study describes the clinical and epidemiological characteristics, treatment, tolerability and outcomes of a cohort of patients with culture-confirmed sporotrichosis treated at a quaternary university hospital in Belo Horizonte and compares localized cutaneous disease with deep infection.

## 2. Materials and Methods

### 2.1. Study Design and Setting

This was a retrospective observational cohort of patients with sporotrichosis treated at the hospital complex of Hospital das Clínicas, Universidade Federal de Minas Gerais (HC-UFMG), in Belo Horizonte, Minas Gerais, Brazil.

HC-UFMG is a quaternary university hospital with more than 400 beds, comprising a complex of dedicated units and outpatient clinics that includes a hospital devoted to ophthalmology and otorhinolaryngology, with a 24-hour ophthalmology emergency service, as well as dermatology, oncology and hematology services and the Orestes Diniz Training and Referral Center for Infectious and Parasitic Diseases. As a state referral center for high-complexity care, it treats patients exclusively through the Brazilian Unified Health System, including patients immunosuppressed by onco-hematological diseases and those in solid organ and hematopoietic stem cell transplant programs. The presence of a dedicated ophthalmology hospital explains the high proportion of ocular cases, and the presence of transplant and oncology services broadens the spectrum of hosts compared with services dedicated exclusively to infectious diseases.

Patients were treated predominantly on an outpatient basis in the infectious disease, dermatology and ophthalmology clinics of the complex, with hospital admission reserved for severe forms.

Patients were identified from the mycology laboratory database and hospital diagnostic codes, and their clinical data were extracted from HC-UFMG medical records. A subgroup of patients at the infectious diseases outpatient clinic have been followed prospectively since 2016, with a structured protocol for clinical and laboratory data collection.

### 2.2. Study Population and Inclusion Criteria

All the patients with a diagnosis of sporotrichosis recorded in the database were considered eligible. The analysis population was restricted to patients with a positive culture for *Sporothrix* spp. Patients with a clinical and epidemiological diagnosis without culture confirmation were not included in this analysis. This criterion corresponds to the definition of a laboratory-confirmed case adopted by the Brazilian Ministry of Health, which requires a positive culture or polymerase chain reaction for *Sporothrix* spp. [21]. Data were recorded in an electronic form in REDCap [28], hosted by the telehealth service of HC-UFMG. No patient without a positive culture had a positive polymerase chain reaction, so the inclusion criterion adopted coincided, in this case series, with the definition of a laboratory-confirmed case. The study period extended from 2011 to 8 August 2026, when the database was closed.

### 2.3. Definitions

Clinical form was classified as fixed cutaneous, lymphocutaneous, disseminated cutaneous, ocular, osteoarticular, pulmonary or disseminated [29]. In the description of baseline characteristics the forms are presented in a non-exclusive manner, and patients with more than one recorded form appear in each corresponding category. For the comparison between groups, localized cutaneous disease comprised the fixed cutaneous and lymphocutaneous forms, and deep infection comprised the disseminated, osteoarticular, pulmonary and disseminated cutaneous forms. Patients with more than one recorded form were allocated according to the most severe form following the hierarchy deep infection, localized cutaneous disease, and ocular disease; accordingly, patients with ocular disease combined with a deep form were allocated to the deep infection group, and patients with ocular disease combined with a localized cutaneous form were allocated to the localized cutaneous group. Patients with isolated ocular disease were not included in either group as they constitute a distinct clinical entity with its own diagnosis, management and outcome and will be described in a separate study. Species identification was performed by polymerase chain reaction in a subgroup of isolates according to a previously described protocol [30]. The primary outcome was clinical cure, defined as complete epithelialization of all the lesions recorded by the attending physician or, in the absence of such a record, completion of antifungal treatment with documented start and end dates. In the latter situation, patients who died, who had the antifungal discontinued for any reason, who remained on chronic suppressive therapy or on treatment at database closure, and those with recorded loss to follow-up were excluded. The service protocol includes follow-up for two months after treatment discontinuation to check for relapse, but this return visit was not documented for all the patients. Outcomes were classified into mutually exclusive categories: cure, spontaneous cure without treatment, treatment failure, sporotrichosis-related death, unrelated death, chronic suppressive therapy, ongoing treatment at database closure, loss to follow-up and no recorded outcome. The relationship between death and sporotrichosis was defined by medical record review. Patients who never received antifungal treatment were not classified as treatment failures. The cure proportion was calculated only among patients with a defined outcome; patients without a defined outcome were excluded from the denominator. Laboratory tests requested during treatment were considered, including aminotransferases, alkaline phosphatase, gamma-glutamyl transferase, bilirubin, amylase, lipase, creatinine and complete blood count. The highest value of each analyte during treatment was recorded. Aminotransferase elevation was graded retrospectively according to the Common Terminology Criteria for Adverse Events, version 5.0 [31], using the upper limit of normal of the local laboratory (aspartate aminotransferase 40 U/L and alanine aminotransferase 41 U/L) and the higher of the two ratios for each patient. The database did not record laboratory tests performed before the start of treatment, so grading assumes a normal baseline value and the observed abnormalities cannot be attributed with certainty to the antifungal agent.

### 2.4. Statistical Analysis

Categorical variables were described as absolute frequencies and proportions and continuous variables as median and interquartile range. Proportions were calculated over the whole study population, with missing data presented as a separate category. Continuous variables were compared using the Mann–Whitney U test and categorical variables using the chi-square or Fisher’s exact test. Binary logistic regression models adjusted for age and sex were built for two outcomes: deep infection and laboratory abnormality during treatment. Comorbidity and immunosuppressive condition were analyzed in separate models because of conceptual overlap. The results are presented as odds ratios with 95% confidence intervals. No model was built for cure or death given the insufficient number of unfavorable outcomes. The analyses were performed in Python 3.12.3 using the pandas 3.0.2, numpy 2.4.4, scipy 1.17.1 and statsmodels 0.14.6 libraries.

### 2.5. Ethics

The study was approved by the Research Ethics Committee of Universidade Federal de Minas Gerais (CAAE 76717223.4.0000.5149). Informed consent was waived for the retrospective data collection. Patients followed prospectively at the infectious disease outpatient clinic since 2016 signed an informed consent form.

## 3. Results

**Figure 1:**
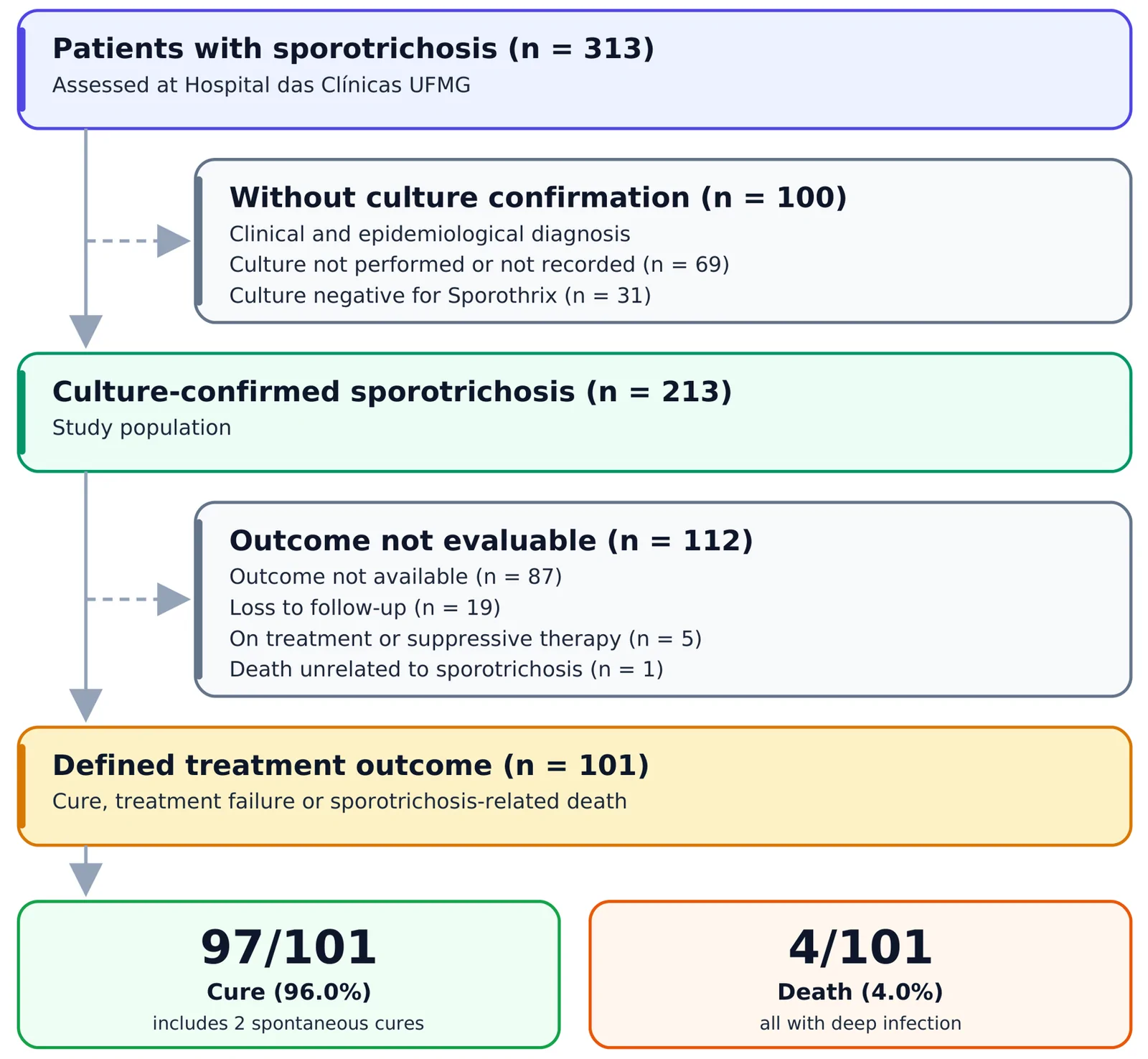
Study flow chart. Patients with sporotrichosis assessed during the study period, showing the culture-confirmed population and the subgroup with a defined outcome. Patients without culture confirmation had a clinical and epidemiological diagnosis of sporotrichosis and are not included in this analysis.

A total of 313 patients with sporotrichosis confirmed by clinical and epidemiological criteria were identified, of whom 213 also had laboratory confirmation by a positive culture for *Sporothrix* spp. and constitute the study population. Among the 100 patients without culture confirmation, 69 had no culture performed or no recorded result and 31 had a negative culture (Figure 1).

### 3.1. Patient Characteristics

The baseline characteristics are shown in Table 1. Of the 213 patients, 115 were female (54.0%). The median age was 41 years (IQR 24 to 55). At least one comorbidity was recorded in 86 patients (40.4%). An immunosuppressive condition was found in 15 (7.0%), often rheumatological disease. The lymphocutaneous form predominated, affecting 115 patients (54.0%). Contact with cats was reported by 113 patients (53.1%), and the animal was ill in 84 of these exposures (74.3%). Species identification was performed in 11 isolates, all *Sporothrix brasiliensis*.

### 3.2. Treatments, Tolerability and Outcomes

The treatments, tolerability and outcomes are shown in Table 2. The most frequently recorded regimen was itraconazole 100 mg twice daily, in 53 patients (24.9%), and amphotericin B was used in 6 (2.8%). The median treatment duration was 150 days (IQR 93 to 217). Laboratory abnormality during treatment was recorded in 52 patients (24.4%) and antifungal discontinuation for any reason in 11 (5.2%). No case was classified as treatment failure. Among the 101 patients with a defined outcome, 97 were cured (96.0%). All four sporotrichosis-related deaths occurred in the deep infection group. One patient died from complications of central nervous system involvement. Another had disseminated disease, never received treatment and was diagnosed after death by blood culture and autopsy. A third, a 36-year-old man with alcohol use disorder, was admitted with disseminated skin lesions of six months’ duration and neurological manifestations and received empirical amphotericin B deoxycholate for eight days, with a cumulative dose of 400 mg, before diagnostic confirmation; autopsy demonstrated meningeal, cerebral, pulmonary, renal, testicular, prostatic and adrenal involvement [32]. The fourth patient, a 52-year-old woman with lymphocutaneous disease that progressed to presumed disseminated infection, with fever, weight loss and concomitant *Staphylococcus aureus* infection, received amphotericin B lipid complex followed by itraconazole and died three weeks after the start of antifungal treatment.

### 3.3. Laboratory Monitoring and Toxicity

The presence or absence of laboratory abnormality during treatment was recorded in 92 patients (43.2%), of whom 52 (56.5%) had at least one altered test. The most frequent laboratory alterations were in the complete blood count, in gamma-glutamyl transferase and alkaline phosphatase, above aminotransferase elevation (Table S1). Peak aminotransferase values were available for 101 patients, a group that does not overlap with the 101 patients with a defined outcome. Of these, 83 had no elevation (82.2%) and a single patient had grade 3 elevation according to the Common Terminology Criteria for Adverse Events, version 5.0 [31]. Patients with an altered test were older and had more comorbidities, more disseminated and osteoarticular disease, and twice as many laboratory panels requested, with no difference in documented cure or antifungal discontinuation (Table 3).

### 3.4. Localized Cutaneous Disease and Deep Infection

A total of 146 patients with localized cutaneous disease and 22 with deep infection were compared (Table 4). The 45 patients with isolated ocular disease were not included in this comparison as they constitute a distinct clinical entity and will be described in a separate study. The patients with deep infection were older (median 50 versus 40 years; *p* = 0.024) and had more comorbidities (81.8% versus 40.4%; *p* < 0.001) and more immunosuppression (27.3% versus 5.5%; *p* = 0.004). Women were less frequent in the deep infection group (31.8% versus 54.1%; *p* = 0.051). Deep infection required more intensive treatment, with itraconazole 400 mg daily (*p* = 0.016), amphotericin B in six patients and none in the localized cutaneous group (*p* < 0.001), and hospital admission in 40.9% versus 4.1% (*p* < 0.001). Cure was documented in 76 of 76 patients with localized cutaneous disease and in five of nine with deep infection (*p* < 0.001), and the four sporotrichosis-related deaths and the three patients on chronic suppressive therapy belonged to the deep infection group. The sporotrichosis-related case fatality was 13.3% among the 15 patients with an immunosuppressive condition and 1.0% among the remaining 198 (*p* = 0.026).

In the multivariable logistic regression analysis, adjusted for age and sex, both the presence of at least one comorbidity (OR 9.69; 95% CI 2.52 to 37.23; *p* < 0.001) and an immunosuppressive condition (OR 6.61; 95% CI 1.89 to 23.09; *p* = 0.003) were independently associated with deep infection in separate models since the two variables overlap conceptually. Female sex was associated with lower odds of deep infection (OR 0.28; 95% CI 0.10 to 0.78; *p* = 0.015 in the model with comorbidity), and age was not associated. For the outcome laboratory abnormality, after adjustment for age, sex, comorbidity and deep infection, only the number of laboratory panels requested remained associated (OR 1.43 per additional panel; 95% CI 1.09 to 1.88; *p* = 0.009) (Table 5).

## 4. Discussion

In this cohort of 213 patients with culture-confirmed sporotrichosis, outcome depended on clinical form: cure was documented in all 76 patients with localized cutaneous disease and in five of nine with deep infection (*p* < 0.001), and the four sporotrichosis-related deaths occurred in the deep infection group. In models adjusted for age and sex, deep infection was independently associated with the presence of a comorbidity (OR 9.69; 95% CI 2.52 to 37.23) and an immunosuppressive condition (OR 6.61; 95% CI 1.89 to 23.09), whereas female sex was associated with lower odds of deep infection. Only 31.8% of the patients with deep infection were women. Among those with localized cutaneous disease, 54.1% were women (*p* = 0.051). Prolonged antifungal treatment was well tolerated, with a single case of grade 3 aminotransferase elevation and discontinuation for any reason in 5.2%.

The observed pattern reproduces that described in Brazilian cohorts of the zoonotic epidemic. In Rio de Janeiro, the historical epicenter, cases in humans and animals have been reported since the end of the 1990s, with cats established as the main source and itraconazole as the first-line treatment [29,33]. In Duque de Caxias, a geo-epidemiological cohort of 827 cases showed a predominance of women aged 25 to 59 years and an association with low income and poor sanitation [34]. In Curitiba, 216 cases followed a rising incidence between 2011 and 2022, with lymphocutaneous disease in 63%, fixed cutaneous disease (termed localized cutaneous in that series) in 24% and ocular disease in 10% [23]. In the state of Amazonas, 950 human cases were notified between 2020 and 2023, with animal contact in 77.7% and *S. brasiliensis* in 65.4% of positive cultures [35]. The historical systematic review of Brazilian cases between 1907 and 2020 gathered 10,400 patients, with 55.98% women, lymphocutaneous disease in 56.14%, systemic involvement in 14.34% and a cure rate of 85.83% [36].

Female predominance is a consistent finding in these series, and the municipal surveillance in Belo Horizonte follows the same direction, with 64% of the cases in women and 67% between 20 and 69 years of age [25]. In this cohort the proportion of women was 54.0%, at the lower limit of what is described in the literature. The difference follows from the way the case series was assembled: a predominantly outpatient cohort from a referral service, restricted to culture-confirmed cases, that receives referrals of atypical, ocular and deep forms from across the region. Typical cutaneous cases, in which female predominance is more marked, are largely diagnosed and treated outside tertiary services, often without culture, and are not part of this case series.

The median age of 41 years and the concentration between 18 and 59 years, which accounted for 65.7% of the cohort, are consistent with other Brazilian series in which the disease affects predominantly working-age adults, with a median of 46 years in São Paulo [37] and a concentration between 25 and 59 years in Duque de Caxias [34]. Twenty-nine patients (13.6%) were younger than 18 years, aged 8 to 17 years, and none had deep infection. Contact with cats was reported by 18 of these 29 patients (62.1%), a higher proportion than in the cohort as a whole, consistent with household exposure to infected animals and the recognition of children as a risk group in the zoonotic epidemic [6].

Contact with cats was reported by 113 patients (53.1%), with an ill animal in 84 of the 113 exposures (74.3%). This proportion probably underestimates the true exposure since the information was not recorded in 39.0% of the cases and is lower than that described in Brazilian series: 87.6% in the classic Rio de Janeiro series [33], 96.5% in São Paulo [37] and 77.7% in the state of Amazonas [35]. In that Rio de Janeiro series, a scratch or bite from a cat was reported by 97 of the 178 patients, so direct traumatic inoculation accounted for only part of the reported exposures. In municipal surveillance in Belo Horizonte, cats account for 74.8% of the identified exposures, and scratching is the most frequent mechanism [25]. Transmission is not restricted to traumatic injury. Cats with sporotrichosis may shed *S. brasiliensis* from the front paws even without apparent lesions, from the nasal cavity and through respiratory droplets, which broadens the routes of exposure and includes contamination of the household environment [38]. A recent household outbreak documented clonal transmission from an index cat to three people and a dog, with distinct clinical presentations among contacts [39]. These findings support the interpretation that female predominance and concentration in young adults reflect the role of caregivers of household cats and reinforce that control of the feline source is the most effective preventive strategy [40].

Species identification was performed by polymerase chain reaction in 11 isolates, all *Sporothrix brasiliensis*. In the remaining 202 patients there was no species identification. This matches the national picture in which *S. brasiliensis* accounts for about 85% of the Brazilian isolates, originating in Rio de Janeiro and spreading to at least six states through independent emergence events followed by animal-to-animal and animal-to-human transmission [7], and with the expansion documented in Minas Gerais [20,27]. *S. schenckii* accounts for about 14% of the isolates in the country, with a regional distribution pattern and greater genetic diversity, consistent with sporadic environmental transmission; in Espírito Santo, *S. brasiliensis* predominated in the metropolitan area, whereas *S. schenckii* was more frequent in the mountainous region [5,41]. Since species is related to severity and atypical presentations [11,16], the absence of routine molecular identification is a limitation of clinical care, not only a methodological one. In this cohort it was not possible to assess whether clinical form or outcome varied according to species, and the isolates were not submitted to antifungal susceptibility testing.

This analysis contrasts localized cutaneous disease with deep infection rather than the usual division between cutaneous and extracutaneous disease. Ocular sporotrichosis comprises, in the great majority of cases, adnexal and surface involvement, with granulomatous conjunctivitis, Parinaud oculoglandular syndrome and dacryocystitis, an entity that the literature already explicitly distinguishes from true intraocular infection [17]. A recent series of 25 cases of ocular sporotrichosis in a hyperendemic region of southern Brazil recorded no case of intraocular involvement, with granulomatous conjunctivitis in 56%, Parinaud syndrome in 40% and dacryocystitis with eyelid lesion in 4% [42]. In this cohort there was no case of endophthalmitis with culture confirmation, so the group with ocular disease comprised exclusively surface involvement. True intraocular involvement is rare and would constitute deep infection.

Resolution of ocular surface disease is assessed by specific parameters, such as resolution of conjunctivitis and patency of the lacrimal system, distinct from the cure criteria that are applicable to skin lesions, and is described in this way in dedicated ophthalmological series [43]. For these reasons ocular cases were analyzed separately and will be described in a separate study. The proportion observed in this case series, 22.1%, is higher than that described in Curitiba, where the ocular form represented 10% of 216 cases [23], and reflects the referral profile of the service, which has a dedicated ophthalmology hospital with a 24-hour emergency service, as well as greater ophthalmological diagnostic capacity, a factor that has already been identified as a determinant of how often this form is recognized [42].

At least one comorbidity was recorded in 81.8% of those patients with deep infection versus 40.4% with localized cutaneous disease and an immunosuppressive condition in 27.3% versus 5.5%. Both remained independently associated with deep infection after adjustment for age and sex, with odds ratios of 9.69 (95% CI 2.52 to 37.23) and 6.61 (95% CI 1.89 to 23.09), respectively. Female sex was associated with lower odds of deep infection, a finding that is consistent with the pattern of the zoonotic epidemic in which women predominate among cases but severe forms concentrate in men with comorbidities. The sporotrichosis-related case fatality was 13.3% among those patients with an immunosuppressive condition and 1.0% among the remainder (*p* = 0.026). Unlike what is described in series from infectious disease services, in which immunosuppression results mainly from HIV infection, in this cohort it was predominantly of rheumatological causes, present in nine of the 15 immunosuppressed patients, which reflects the referral profile of a university hospital with all specialties.

This agrees with the Brazilian literature in which immunosuppression, alcohol use and diabetes mellitus are the main determinants of dissemination. In a cohort from the same state, all the patients living with HIV developed the systemic form, with increased mortality, and alcohol use was specifically associated with pulmonary involvement [19]; in the individual participant data review, alcohol use disorder remained independently associated with systemic disease, with an odds ratio of 4.76 (95% CI 1.45 to 18.05) [44]; and in an osteoarticular series from Belo Horizonte diabetes was an independent factor, with an odds ratio of 4.11 [20]. In this cohort none of these isolated factors reached significance: diabetes was not independently associated with deep infection and alcohol use was borderline (*p* = 0.071), with information missing for more than half of the patients. HIV infection was recorded in only two patients (0.9%), a far lower proportion than in series of systemic disease, which reflects both underreporting and the referral profile of the service. What remained associated was the broader category, comorbidity or immunosuppression, which suggests that, in services with a wide spectrum of hosts, the determinant of severity is the presence of immunocompromise itself rather than a specific condition.

From a clinical care perspective, these findings support that the initial assessment of every patient with sporotrichosis should include active search for predisposing conditions, with HIV testing, assessment of blood glucose and history of alcohol use regardless of clinical presentation. Immunosuppression of non-infectious cause, predominant in this case series, tends to be less often considered a risk factor for implantation mycosis than HIV infection. The risk of iatrogenic immunosuppression deserves particular attention: cutaneous sporotrichosis may be mistaken for inflammatory dermatoses, and disseminated disease has been described after corticosteroid therapy started for suspected pyoderma gangrenosum [45]. In this cohort, a patient on methotrexate and prednisone for rheumatoid arthritis had a biopsy that was initially interpreted as pyoderma gangrenosum, with the culture revealing *Sporothrix* spp., which reinforces the need for mycological investigation before intensifying immunosuppression in chronic ulcerated skin lesions.

Cure was documented in all 76 patients with localized cutaneous disease and in five of nine with deep infection, and the four sporotrichosis-related deaths and the three patients maintained on chronic suppressive therapy belong to the deep infection group. The difference in cure according to clinical form is similar to that described in the individual participant data review [44] and observed in a global cohort in which disseminated disease, malignancy and older age were associated with higher one-year mortality [46]. Treatment also reproduced the described pattern: localized cutaneous disease responds to itraconazole at conventional doses for three to six months, whereas deep forms require higher doses or amphotericin B, reserved for systemic cases [17,47]. The number of patients with deep infection and a defined outcome is small, which precludes risk estimation, but the direction of the finding is consistent with series of systemic, osteoarticular and pulmonary disease from different Brazilian states [12,13,16,18,20].

All four sporotrichosis-related deaths occurred in patients with disseminated disease, although dissemination was presumed rather than microbiologically confirmed in one patient, and in two the diagnosis was established only at the time of death or afterwards. One patient never received antifungal treatment, and the diagnosis was made after death by blood culture and autopsy. Further, a 36-year-old man with six months of disseminated skin lesions received empirical amphotericin B deoxycholate and had the biopsy result released one day before death, with the definitive culture report available only after death [32]. A third patient died from complications of central nervous system involvement. Two of the four patients who died had central nervous system involvement, an uncommon complication with high lethality, described predominantly in immunocompromised patients and associated with unfavorable outcomes even under prolonged treatment [15].

These patients reached the service already with disseminated disease and in severe condition after a prolonged course. This database contains no information on symptom onset or the previous care pathway, but the presentation suggests that dissemination resulted from the chronic evolution of untreated disease and not from the interval between admission and the start of treatment. The increase in hospital admissions for sporotrichosis, a disease that is typically managed on an outpatient basis, has been described in other regions of the country: in Pernambuco mycosis became the second leading cause of hospital admission for fungal disease between 2016 and 2024, with an increase from five to 28 annual admissions and disseminated disease in 17.3% of the specified cases [24]. The critical point lies before access to the referral service in the recognition of the disease in its early forms.

With a median treatment duration of 150 days, aminotransferase elevation was restricted to grades 1 and 2 in almost all the patients assessed, with a single case of grade 3, and antifungal discontinuation for any reason occurred in 5.2%. The most frequent abnormalities were in the complete blood count and gamma-glutamyl transferase, non-specific findings, and were more frequent than aminotransferase elevation. The profile is similar to that described in the largest series of treatment of cutaneous sporotrichosis with itraconazole, with 645 patients, in which clinical adverse events occurred in 18.1% of those treated with 100 mg daily and in 21.9% of those receiving 200 to 400 mg daily, predominantly nausea and epigastric pain [48]. Hepatotoxicity is the most relevant adverse effect of prolonged azole use, although it is rare at conventional doses [49].

These data support outpatient management of itraconazole with targeted rather than systematic laboratory monitoring in patients without previous liver or kidney disease and on conventional doses. Regimens recommended for deep infection may involve higher doses and courses exceeding twelve months, which increase cumulative exposure and justify periodic monitoring of liver function tests, particularly in patients with liver comorbidity, concomitant use of other hepatotoxic agents or alcohol consumption [50].

The comparison between patients with and without laboratory abnormalities should be interpreted with caution. The patients with abnormalities were older and had more comorbidities and deep infections, but twice as many laboratory panels were requested, and, in the multivariable analysis, only the number of panels remained associated with the outcome. Part of the difference reflects the intensity of the investigation rather than toxicity. The absence of differences in cure and antifungal discontinuation between the two groups suggests that the identified abnormalities had few clinical consequences.

### Limitations

This is a retrospective cohort, and the data were extracted from records that were not generated for research purposes, so completeness varies across variables. Outcomes were not recorded in 40.8% of the patients, which restricts the cure proportion to a subgroup with documented follow-up and may overestimate sustained cure.

The database did not record laboratory tests performed before the start of treatment, so the observed abnormalities cannot be attributed with certainty to the antifungal agent, and grading assumes a normal baseline value. The laboratory monitoring was not protocol-driven, and its intensity varied according to case severity, which introduces detection bias.

The proportion of patients with HIV infection was 0.9%, far lower than the 15% described among osteoarticular cases from an infectious disease referral service in the same city [20]. Part of this reflects the referral profile: HC-UFMG receives patients from all specialties, and immunosuppression in the cohort is more heterogeneous, including solid organ transplant recipients and patients on immunosuppressive drugs for autoimmune disease, whereas series from hospitals dedicated to infectious diseases concentrate patients living with HIV.

Species identification was performed in only 11 isolates, which precludes any inference about species distribution; Brazilian cohort studies, however, confirm that the great majority of cases in the south and southeast regions are caused by *Sporothrix brasiliensis*. The temporal distribution of cases reflects the case-finding process and should not be read as an epidemic curve. Finally, with 21 events and three variables, the models for deep infection had seven events per variable, below the usual rule of ten, and the confidence intervals are wide.

## 5. Conclusions

In a cohort of 213 patients with culture-confirmed sporotrichosis at a referral center in Belo Horizonte, the disease affected mainly adult women, with lymphocutaneous disease and exposure to ill cats, and cure was documented in 96.0% of those patients with a defined outcome. Prolonged antifungal treatment was well tolerated, with grade 3 aminotransferase elevation in a single patient and antifungal discontinuation in 5.2%. Deep infection was concentrated in older patients with comorbidities, required more intensive regimens and accounted for all the sporotrichosis-related mortality, including two cases diagnosed only at the time of death or afterwards. Early recognition of deep presentations and routine species identification are the two needs identified by this study.

## Figures and Tables

**Table 1:** Baseline characteristics of patients with culture-confirmed sporotrichosis (*n* = 213).

Characteristic	Patients (*n* = 213)
**Sex**	
Female	115 (54.0)
Male	98 (46.0)
**Age, years**	
Median (IQR)	41 (24–55) [*n* = 211]
0–17	29 (13.6)
18–39	72 (33.8)
40–59	68 (31.9)
≥60	42 (19.7)
Not reported	2 (0.9)
**Self-reported skin color**	
Mixed race (parda)	133 (62.4)
White	22 (10.3)
Black	14 (6.6)
Asian	3 (1.4)
Not reported	41 (19.2)
**Place of residence**	
Belo Horizonte	107 (50.2)
Metropolitan Region of Belo Horizonte	54 (25.4)
Interior of Minas Gerais	16 (7.5)
Other Brazilian states	1 (0.5)
Not reported	35 (16.4)
**Comorbidities**	
At least one comorbidity	86 (40.4)
Hypertension	42 (19.7)
Diabetes mellitus	16 (7.5)
Smoking	15 (7.0)
Depression or anxiety	12 (5.6)
Obesity (BMI ≥ 30)	7 (3.3)
Chronic kidney disease	8 (3.8)
Heart disease	4 (1.9)
Hypothyroidism	5 (2.3)
Asthma or COPD	6 (2.8)
Liver disease	4 (1.9)
Other psychiatric disorder	5 (2.3)
Other comorbidity	27 (12.7)
**Immunosuppressive condition**	
Any immunosuppressive condition	15 (7.0)
Rheumatological disease	9 (4.2)
HIV infection	2 (0.9)
Inflammatory bowel disease	1 (0.5)
Glomerulopathy	1 (0.5)
Solid organ transplant	1 (0.5)
Intracranial tumour	1 (0.5)
**Clinical form**	
Lymphocutaneous	115 (54.0)
Fixed cutaneous	44 (20.7)
Ocular	47 (22.1)
Disseminated	13 (6.1)
Osteoarticular	12 (5.6)
Disseminated cutaneous	3 (1.4)
Pulmonary	2 (0.9)
More than one form recorded	19 (8.9)
**Hospital admission**	
Hospital admission recorded	16 (7.5)
**Site of involvement**	
Upper limbs	102 (47.9)
Lower limbs	41 (19.2)
Face	11 (5.2)
Trunk	11 (5.2)
Ocular	36 (16.9)
Neck	7 (3.3)
Nose	4 (1.9)
Mouth	2 (0.9)
Not reported	47 (22.1)
**Exposure**	
Contact with cats	113 (53.1)
Not reported (cat contact)	83 (39.0)
Sick cat among those exposed to cats	84/113 (74.3)
Contact with animals other than cats	23 (10.8)
Contact with soil, gardening	21 (9.9)
Not reported (soil contact)	130 (61.0)
Any animal exposure	119 (55.9)
**Mycological diagnosis**	
*Sporothrix* spp., not identified to species	202 (94.8)
*Sporothrix brasiliensis* (molecular identification)	11 (5.2)

Data are n (%) unless otherwise stated. IQR, interquartile range. HIV infection is reported once under immunosuppressive condition. Categories of comorbidity are not mutually exclusive, and patients may appear in more than one row. “Other comorbidity” comprises conditions entered as free text, most frequently dyslipidemia (*n* = 5), rhinitis or allergy (*n* = 5), gastrointestinal disease (*n* = 4), malignancy (*n* = 3), thrombosis or coagulation disorder (*n* = 3) and epilepsy (*n* = 2); rheumatological disease is shown in its own row under immunosuppressive condition. Bold indicates category headings.

**Table 2:** Treatments, tolerability and outcomes of patients with culture-confirmed sporotrichosis (*n* = 213).

Variable	Patients (*n* = 213)
**Treatment received**	
Itraconazole 100 mg twice daily	53 (24.9)
Itraconazole 200 mg once daily	44 (20.7)
Itraconazole 400 mg daily	21 (9.9)
Potassium iodide	13 (6.1)
Terbinafine	5 (2.3)
Amphotericin B deoxycholate	3 (1.4)
Liposomal amphotericin B	1 (0.5)
Amphotericin B lipid complex	4 (1.9)
Amphotericin B, any formulation	6 (2.8)
Local heat	1 (0.5)
Other	16 (7.5)
More than one regimen recorded	22 (10.3)
No antifungal treatment	3 (1.4)
Treatment not recorded	75 (35.2)
**Treatment duration**	
Median (IQR), days	150 (93–217)
Available, n (%)	106 (49.8)
**Tolerability**	
Laboratory abnormality during treatment	52 (24.4)
Antifungal discontinuation for any reason	11 (5.2)
**Outcome**	
Cure after antifungal treatment	95 (44.6)
Spontaneous cure without treatment	2 (0.9)
Treatment failure	0 (0.0)
Death, sporotrichosis-related	4 (1.9)
Death, unrelated to sporotrichosis	1 (0.5)
Chronic suppressive therapy	3 (1.4)
On treatment at database closure	2 (0.9)
Loss to follow-up	19 (8.9)
No outcome recorded	87 (40.8)
Overall cure among patients with a defined outcome	97/101 (96.0)

Data are n (%) unless otherwise stated. IQR, interquartile range. Treatment categories are not mutually exclusive: 22 patients received more than one regimen, and the six patients treated with amphotericin B are also counted under the individual formulations received. Bold indicates category headings.

**Table 3:** Comparison of patients with and without laboratory abnormalities during antifungal treatment (*n* = 92).

Variable	Laboratory Abnormality (*n* = 52)	No Abnormality (*n* = 40)	*p* Value
**Demographic characteristics**			
Age, years, median (IQR)	50 (38–56) [*n* = 52]	36 (20–47) [*n* = 40]	0.003
Age ≥ 60 years	11 (21.2)	6 (15.0)	0.451
Female sex	29 (55.8)	26 (65.0)	0.371
**Comorbidities**			
At least one comorbidity	38 (73.1)	20 (50.0)	0.023
Hypertension	19 (36.5)	9 (22.5)	0.147
Diabetes mellitus	7 (13.5)	3 (7.5)	0.505
Liver disease	1 (1.9)	1 (2.5)	1.000
Chronic kidney disease	4 (7.7)	1 (2.5)	0.383
Obesity (BMI ≥ 30)	4 (7.7)	1 (2.5)	0.383
**Clinical form**			
Lymphocutaneous	27 (51.9)	23 (57.5)	0.594
Fixed cutaneous	13 (25.0)	11 (27.5)	0.787
Ocular	7 (13.5)	8 (20.0)	0.400
Disseminated	8 (15.4)	0 (0.0)	0.009
Osteoarticular	7 (13.5)	0 (0.0)	0.017
**Antifungal regimen**			
Itraconazole 100 mg twice daily	24 (46.2)	16 (40.0)	0.555
Itraconazole 200 mg once daily	14 (26.9)	19 (47.5)	0.041
Itraconazole 400 mg daily	11 (21.2)	3 (7.5)	0.085
Amphotericin B, any formulation	1 (1.9)	0 (0.0)	1.000
Potassium iodide	1 (1.9)	0 (0.0)	1.000
More than one regimen recorded	9 (17.3)	3 (7.5)	0.219
**Treatment and monitoring**			
Treatment duration, days, median (IQR)	162 (94–304) [*n* = 46]	122 (90–176) [*n* = 30]	0.068
Test panels requested, median (IQR)	4 (2–6) [*n* = 49]	2 (1–2) [*n* = 39]	<0.001
**Outcome**			
Documented cure	37 (71.2)	29 (72.5)	0.887
Loss to follow-up	4 (7.7)	5 (12.5)	0.495
Death, any cause	2 (3.8)	0 (0.0)	0.503
Antifungal discontinuation	4 (7.7)	2 (5.0)	0.694

IQR, interquartile range. The analysis is restricted to patients regarding whom the presence or absence of laboratory abnormality during treatment was recorded. Documented cure is calculated over the whole group in each column and not over the patients with a defined outcome. Numbers in square brackets indicate the patients with available data for the variable. Bold indicates category headings.

**Table 4:** Comparison of localized cutaneous disease and deep infection (*n* = 168).

Variable	Localized Cutaneous (*n* = 146)	Deep Infection (*n* = 22)	*p* Value
**Demographic characteristics**			
Age, years, median (IQR)	40 (25–56) [*n* = 141]	50 (43–62) [*n* = 21]	0.024
Age ≥ 60 years	30 (20.5)	6 (27.3)	0.577
Female sex	79 (54.1)	7 (31.8)	0.051
**Comorbidities**			
At least one comorbidity	59 (40.4)	18 (81.8)	<0.001
Hypertension	35 (24.0)	5 (22.7)	0.898
Diabetes mellitus	12 (8.2)	4 (18.2)	0.232
HIV infection	1 (0.7)	1 (4.5)	0.245
Chronic kidney disease	4 (2.7)	2 (9.1)	0.177
Immunosuppressive condition	8 (5.5)	6 (27.3)	0.004
Alcohol use among those with information	13/66 (19.7)	6/13 (46.2)	0.071
**Hospital admission**			
Hospital admission recorded	6 (4.1)	9 (40.9)	<0.001
**Exposure**			
Contact with cats	86 (58.9)	10 (45.5)	0.235
Sick cat among those exposed to cats	67/86 (77.9)	6/10 (60.0)	0.245
Contact with soil or gardening	15 (10.3)	3 (13.6)	0.710
**Antifungal treatment**			
Itraconazole 100 mg twice daily	42 (28.8)	3 (13.6)	0.135
Itraconazole 200 mg once daily	33 (22.6)	4 (18.2)	0.787
Itraconazole 400 mg daily	12 (8.2)	6 (27.3)	0.016
Potassium iodide	11 (7.5)	1 (4.5)	1.000
Terbinafine	5 (3.4)	0 (0.0)	1.000
Amphotericin B, any formulation	0 (0.0)	6 (27.3)	<0.001
More than one regimen recorded	15 (10.3)	5 (22.7)	0.147
Treatment duration, days, median (IQR)	150 (96–216) [*n* = 78]	98 (44–331) [*n* = 12]	0.330
**Tolerability**			
Laboratory abnormality if monitored	36/67 (53.7)	11/12 (91.7)	0.022
Antifungal discontinuation	9 (6.2)	2 (9.1)	0.639
**Outcome**			
Cure among defined outcomes	76/76 (100.0)	5/9 (55.6)	<0.001
Death, sporotrichosis-related	0 (0.0)	4 (18.2)	<0.001
Chronic suppressive therapy	0 (0.0)	3 (13.6)	0.002
Loss to follow-up	15 (10.3)	1 (4.5)	0.698
No outcome recorded	52 (35.6)	9 (40.9)	0.630

IQR, interquartile range. Localized cutaneous disease includes the fixed cutaneous and lymphocutaneous forms. Deep infection includes the disseminated, osteoarticular, pulmonary and disseminated cutaneous forms. Patients with more than one recorded clinical form were allocated to a single group according to the most severe form, so the group totals do not correspond to the sum of the non-exclusive clinical form counts shown in Table 1. Numbers in square brackets indicate the patients with available data for the variable. Bold indicates category headings.

**Table 5:** Multivariable logistic regression for deep infection and laboratory abnormality during treatment.

Variable	Odds Ratio	95% CI	*p* Value
**Deep infection: any comorbidity as exposure (** ***n*** **= 162, with 21 events)**			
Age, per 10 years	1.10	0.84–1.45	0.475
Female sex	0.28	0.10–0.78	0.015
At least one comorbidity	9.69	2.52–37.23	<0.001
**Deep infection: immunosuppressive condition as exposure (** ***n*** **= 162, with 21 events)**			
Age, per 10 years	1.24	0.96–1.61	0.100
Female sex	0.36	0.13–1.01	0.051
Immunosuppressive condition	6.61	1.89–23.09	0.003
**Laboratory abnormality during treatment (** ***n*** **= 88, with 49 events)**			
Age, per 10 years	1.23	0.92–1.62	0.157
Female sex	0.71	0.26–1.91	0.493
At least one comorbidity	0.92	0.30–2.83	0.890
Deep infection	2.60	0.24–27.61	0.428
Number of test panels requested	1.43	1.09–1.88	0.009

CI, confidence interval. Binary logistic regression models estimated by maximum likelihood, with Wald confidence intervals. Sample sizes are smaller than the corresponding group totals because patients with missing covariate data were excluded from each model (complete-case analysis): 162 of the 168 patients compared in Table 4 had age recorded, and 88 of the 92 patients in Table 3 had the number of laboratory panels recorded. Bold indicates category headings.

## Data Availability

The data presented in this study are available on request from the corresponding author. The data are not publicly available because they contain information that could compromise the privacy of research participants.

## Supplementary Materials

The following supporting information can be downloaded at https://www.mdpi.com/article/10.3390/jof12090685/s1, Table S1: Laboratory monitoring, toxicity and antifungal discontinuation (*n* = 213).
