# Supplementary material for: Localized Cutaneous Versus Deep Sporotrichosis at Hospital das Clínicas, UFMG, Belo Horizonte, Brazil: A Cohort of 213 Culture-Confirmed Cases, 2011 to 2026
Source: J Fungi (Basel). 2026 Sep 11;12(9):685. doi: 10.3390/jof12090685 (PMC13608755; doi:10.3390/jof12090685)
Supplement: Supplementary file 1 [file jof-12-00685-s001.zip › jof-4523110-supplementary.pdf]

Supplementary Materials

Table S1. Laboratory monitoring, toxicity and antifungal discontinuation in patients with culture-confirmed sporotrichosis (n = 213).

Variable	Patients (n = 213)
**Laboratory monitoring**	
Laboratory monitoring requested	93 (43.7)
Not recorded	101 (47.4)
Number of test panels requested, median (IQR)	2 (1–4) [n=113]
**Laboratory abnormality during treatment**	
Any abnormality	52 (24.4)
No abnormality	40 (18.8)
Not recorded	121 (56.8)
**Abnormal test, among all patients**	
Aspartate aminotransferase	8 (3.8)
Alanine aminotransferase	11 (5.2)
Alkaline phosphatase	18 (8.5)
Gamma-glutamyl transferase	25 (11.7)
Amylase	5 (2.3)
Lipase	8 (3.8)
Creatinine	11 (5.2)
Complete blood count	27 (12.7)
Other	16 (7.5)
**Peak value during treatment**	
Aspartate aminotransferase, U/L	25 (20–31) [n=101]
Alanine aminotransferase, U/L	22 (17–32) [n=100]
Alkaline phosphatase, U/L	86 (67–120) [n=82]
Gamma-glutamyl transferase, U/L	35 (19–67) [n=86]
Total bilirubin, mg/dL	0.60 (0.45–0.79) [n=76]
Direct bilirubin, mg/dL	0.22 (0.13–0.36) [n=67]
Amylase, U/L	77 (57–102) [n=38]
Lipase, U/L	88 (59–152) [n=38]
Creatinine, mg/dL	0.78 (0.67–1.00) [n=83]
**Peak aminotransferase elevation (CTCAE grade)**	
No elevation (≤ upper limit of normal)	83/101 (82.2)
Grade 1 (>1 to 3× upper limit of normal)	13/101 (12.9)
Grade 2 (>3 to 5× upper limit of normal)	4/101 (4.0)
Grade 3 or higher (>5× upper limit of normal)	1/101 (1.0)
Total bilirubin above 1.5 mg/dL	2/76 (2.6)
Creatinine above 1.3 mg/dL	9/83 (10.8)
**Antifungal discontinuation**	
Discontinued for any reason	11 (5.2)
Not recorded	108 (50.7)
**Reason for discontinuation, among all patients**	
Hepatotoxicity	1 (0.5)
Renal dysfunction	3 (1.4)

Cutaneous reaction or allergy	3 (1.4)
Pancreatic enzyme elevation	0 (0.0)
Drug interaction	2 (0.9)
Pregnancy	1 (0.5)
Patient decision or misunderstanding	1 (0.5)

Data are n (%) unless otherwise stated. IQR, interquartile range; CTCAE, Common Terminology Criteria for Adverse Events. Peak values are the highest value recorded during treatment, and the number of patients with an available result is shown for each analyte. Aminotransferase elevation was graded retrospectively according to CTCAE version 5.0, using the upper limit of normal of the local laboratory (aspartate aminotransferase 40 U/L and alanine aminotransferase 41 U/L) and the higher of the two ratios for each patient. Abnormal tests and reasons for discontinuation are not mutually exclusive. Laboratory tests performed before the start of treatment were not recorded in the database.
